# Crystal structure of (1*Z*)-1-(4-chloro­benzyl­idene)-5-(4-meth­oxy­phen­yl)-3-oxopyrazolidin-1-ium-2-ide

**DOI:** 10.1107/S1600536814014445

**Published:** 2014-07-19

**Authors:** Peter Mangwala Kimpende, Thi Kieu Oanh Doan, Quoc Trung Vu, Luc Van Meervelt

**Affiliations:** a Chemistry Department, University of Kinshasa, Kinshasa XI BP 212, Democratic Republic of the Congo; b Faculty of Chemistry, Hanoi National University of Education, 136 – Xuan Thuy – Cau Giay, Hanoi, Vietnam; c Chemistry Department, KU Leuven, Celestijnenlaan 200F, B-3001 Leuven (Heverlee), Belgium

**Keywords:** crystal structure, pyrazolidinium ylide, betaine structure

## Abstract

The title mol­ecule, C_17_H_15_ClN_2_O_2_, is L-shaped with the 4-chloro­benzyl­idene ring almost coplanar with the planar pyrazolidine ring (r.m.s. deviation = 0.020 Å), making a dihedral angle of 4.83 (17)°. The 4-meth­oxy­phenyl ring is almost normal to the mean plane of the pyrazolidine ring and the 4-chloro­benzyl­idene ring, with dihedral angles of 87.36 (17) and 89.23 (16)°, respectively. The pyrazolidine ring occurs in the betaine form with a *Z* configuration for the exocyclic C=N bond. In the crystal, C—H⋯O and C—H⋯π inter­actions generate ribbons of mol­ecules along [1-10].

## Abstract 2

The planar pyrazolidine ring occurs in the betaine form with a *Z* conformation of the exocyclic C=N bond. In the crystal, C—H⋯O and C—H⋯π inter­actions result in the formation of ribbons of mol­ecules along [1
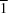
0].

## Chemical context   

Acyclic azomethine imides are difficult to synthesize and have thus rarely been explored. However, cyclic azomethine imides of the 3-oxopyrazolidin-1-um-2-ide type are generated under mild conditions and have largely been used for the novel synthesis of heterocyclic compounds (Schantl, 2004 ▶; Padwa & Pearson, 2003 ▶) such as monocyclic and bicyclic pyrazolidin­­ones (Zhou *et al.*, 2013 ▶; Suarez *et al.*, 2005 ▶) and other bicyclic heterocycles (Svete, 2006 ▶; Xu *et al.*, 2013 ▶). Since numerous pyrazole derivatives have found use in pharmaceutical, agrochemical and other applications, for example, sildenafil or Viagra (Mulhall, 1997 ▶), lonazolac (Vinge & Bjorkman, 1986 ▶), merpirizole (Naito *et al.*, 1969 ▶), the bicyclic pyrazolidinone LY 186826 (Indelicato & Pasini, 1988 ▶) and the developing agent in photography, phenidone, a part of our studies is focused on the synthesis of functionalized pyrazoles. For this purpose, the title compound was synthesized and the mol­ecular and crystal structure are reported herein.
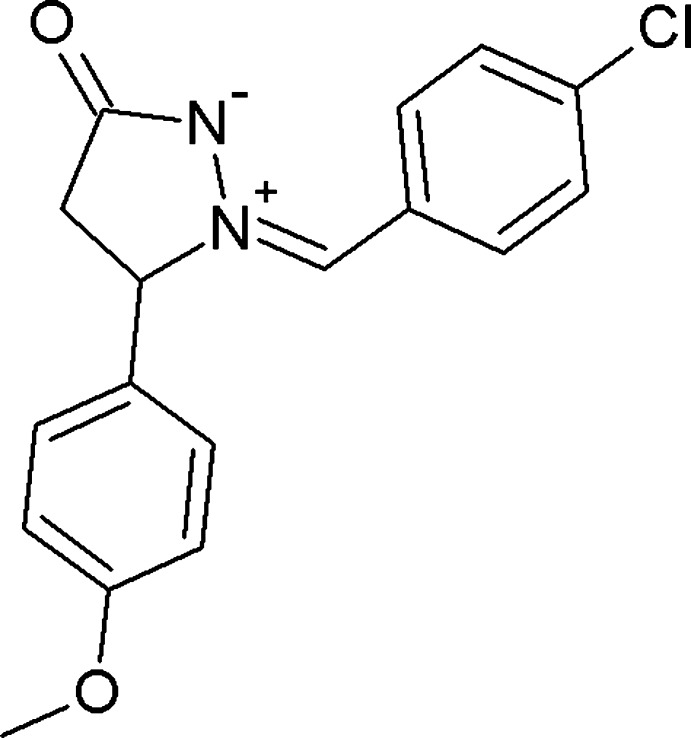


## Structural Commentary   

**Figure 1:**
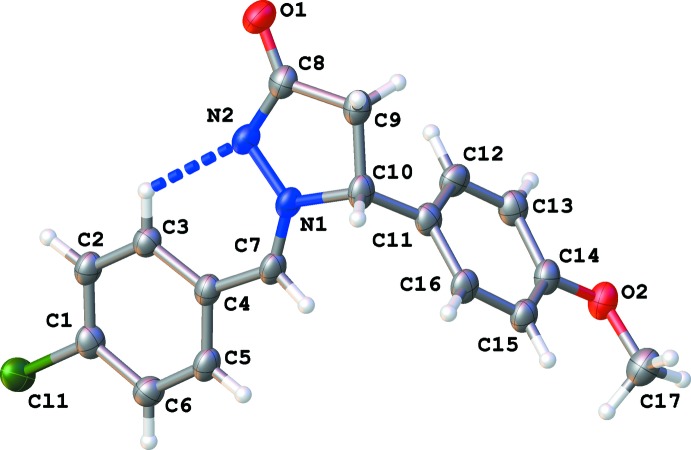
Mol­ecular structure of the title mol­ecule, with atom labelling. Displacement ellipsoids are drawn at the 50% probability level. The intra­molecular C—H⋯N inter­action is drawn as a dashed line (see Table 1 ▶ for details).

The pyrazolidine ring is planar with a maximal deviation of 0.017 (3) Å for atom C10. The 4-chloro­benzyl aromatic ring and the pyrazolidine ring are almost coplanar, making a dihedral angle of 4.83 (17)°, whereas the mean plane through the 4-meth­oxy­phenyl aromatic ring is almost perpendicular [87.36 (17)°] to the pyrazolidine plane. The aromatic rings are inclined to one another at 89.23 (16)°. The configuration of the exocyclic C1=N7 bond is *Z*. The pyrazolidine ring shows a betaine character with opposite charges located on adjacent nitro­gen atoms, N1 and N2. The N1—N2 bond distance of 1.362 (3) Å agrees with the average value of 1.357 (7) Å obtained for N^+^—N^−^ in pyrazolidine rings found in the Cambridge Structural Database (CSD, Version 5.35, February 2014; Allen, 2002 ▶). The intra­molecular C3—H3⋯N2 inter­action (Table 1 ▶ and Fig. 1 ▶) is also observed in similar compounds found in the CSD.

## Supra­molecular features   

**Figure 2:**
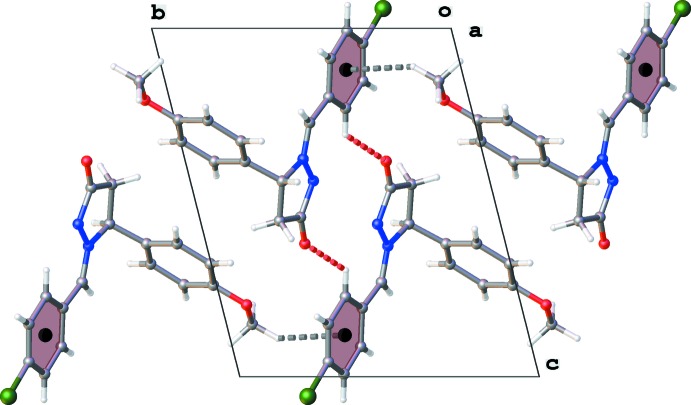
Crystal packing for the title compound viewed along the *a* axis, with the C—H⋯π and C—H⋯O inter­actions drawn as dashed lines (see Table 1 ▶ for details).

In the crystal packing C–H⋯O hydrogen bonds are observed (Table 1 ▶ and Fig. 2 ▶), resulting in the formation of inversion dimers with 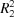
(16) loops. Furthermore, the aromatic ring of the 4-chloro­benzyl substituent is involved in C—H⋯π inter­actions (Table 1 ▶ and Fig. 2 ▶), forming ribbons of dimers propagating along [1
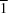
0].

## Database survey   

The Cambridge Structural Database contains 15 crystal structures containing a similar 1-methyl­idene-3-oxopyrazol­idin-1-ium-2-ide fragment. For the 12 structures bearing a 1-benzyl­idene substituent, the dihedral angle between its aromatic ring and the pyrazolidine ring varies from 0.0 to 65.6° depending on the further substitution of the 1-benzyl­idene substituent. A fit of the common parts of the title compound and (1*Z*)-1-(4-chloro­benzyl­idene-5,5-dimethyl-3-oxopyrazol­idin-1-ium-2-ide (refcode: BOLJUH; Kulpe *et al.*, 1983 ▶) results in an r.m.s. deviation of 0.069 Å.

## Synthesis and crystallization   

**Figure 3:**
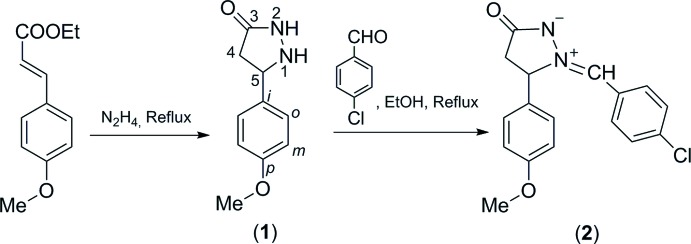
Reaction scheme for the title compound.

The starting material, ethyl *p*-meth­oxy­cinnamate, was isolated from *Kaempferia galanga* L., a traditional medicinal plant in Vietnam (Do, 2011 ▶). The reaction scheme to synthesize the title compound, (2), is given in Fig. 3 ▶.

**Synthesis of 5-** ***p*** **-meth­oxy­phenyl­pyrazolidin-3-one (1)**: A solution of 1.03 g (5 mmol) of ethyl *p*-meth­oxy­cinnamate, 0.5 ml of N_2_H_4_·H_2_O 80% in 5 ml of ethanol was refluxed for 24 h. To the cool mixture 0.2 ml of H_2_O was added and allowed to stand. The resulting precipitate was collected and recrystallized from ethanol to give 0.54 g (yield 56%) of (1) in the form of white crystals; m.p. 442–443 K. IR (KBr, cm^−1^): 3229, 3180 (NH); 3041, 2951, 2834 (C—H), 1675 (C=O); 1605, 1520 (phenyl C=C). ^1^H NMR (*d*_6_-DMSO, δ, ppm; *J*, Hz): 9.14 *s* (N^2^H); 5.46 broadened *s*, (N^1^H); 2.63 *dd*, ^2^ *J* 15.5, ^3^ *J* 7.5 (H^4a^); 2.37 *dd*, ^2^ *J* 15.5, ^3^ *J* 8.0 (H^4b^); 4.52 *t*, ^3^ *J* 7.5 (H^5^); 7.32 *d*, ^3^ *J* 8.5 (2H, H^*o*^); 6.91 *d*, ^3^ *J* 8.5 (2H, H^*m*^); 3.74 *s* (3H, MeO). ^13^C NMR [*d*_6_-DMSO, δ, p.p.m., according to the HSQC and HMBC spectra of (1)]: 175.37 (C^3^), 39.00 (C^4^), 59.87 (C^5^), 132.37 (C^*i*^), 127.85 (C^*o*^), 113.66 (C^*m*^), 158.51 (C^*p*^), 55.06 (MeO). Analysis*:* calculated for C_10_H_12_N_2_O_2_: C, 62.49; H, 6.29; N, 14.57; found: C, 62.71; H, 6.08; N, 14.29.

**Synthesis of 1-(** ***p*** **-chloro­benzyl­idene)-5-(*p*-meth­oxy­phen­yl)-3-oxopyrazolidin-1-ium-2-ide (2)**: A solution of 0.192 g (1 mmol) of (1) and 0.141 g (1 mmol) of 4-chloro­benzaldehyde in 5 ml of ethanol was refluxed for 6 h. The reaction mixture was allowed to cool. The resulting precipitate was collected and recrystallized from ethanol to give 0.22 g (yield 70%) of (2) as white crystals; m.p. 467–468 K. IR (KBr, cm^−1^): 3095, 3052, 2930, 2852 (C-H), 1676 (C=O); 1587, 1563, 1512 (phenyl C=C). Analysis*:* calculated for C_17_H_15_ClN_2_O_2_: C, 64.87; H, 4.80; N, 8.90. Found: C, 65.08; H, 4.59; N, 8.64.

Colourless plate-like crystals of (2) suitable for X-ray diffraction were obtained by slow evaporation from a water solution acidified with HCl at room temperature.

## Refinement   

Crystal data, data collection and structure refinement details are summarized in Table 2 ▶. All H atoms were refined using a riding model with stretchable C—H distances, and with *U*_iso_ = 1.5*U*_eq_(C-meth­yl) and = 1.2*U*_eq_(C) for other H atoms.

## Supplementary Material

Crystal structure: contains datablock(s) 2. DOI: 10.1107/S1600536814014445/su0007sup1.cif

Structure factors: contains datablock(s) 2. DOI: 10.1107/S1600536814014445/su00072sup2.hkl

Supporting information file. DOI: 10.1107/S1600536814014445/su00072sup3.cml

CCDC reference: 1006910

Additional supporting information:  crystallographic information; 3D view; checkCIF report

## Figures and Tables

**Table 1:** Hydrogen-bond geometry (Å, °) *Cg* is the centroid of the C1–C6 ring.

*D*—H⋯*A*	*D*—H	H⋯*A*	*D*⋯*A*	*D*—H⋯*A*
C3—H3⋯N2	0.96 (4)	2.31 (3)	2.934 (4)	122 (1)
C3—H3⋯O1^i^	0.96 (4)	2.52 (2)	3.152 (4)	124 (1)
C17—H17*C*⋯*Cg* ^ii^	1.02 (3)	2.73 (3)	3.551 (4)	138 (2)

Symmetry codes: (i) 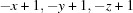; (ii) 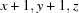.

**Table 2:** Experimental details

Crystal data	
Chemical formula	C_17_H_15_ClN_2_O_2_
*M*_r_	314.76
Crystal system, space group	Triclinic, *P* 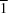
Temperature (K)	100
*a*, *b*, *c* (Å)	5.6966 (6), 10.6852 (13), 12.7750 (17)
α, β, γ (°)	101.573 (7), 100.620 (7), 101.311 (6)
*V* (Å^3^)	726.47 (15)
*Z*	2
Radiation type	Cu *K*α
μ (mm^−1^)	2.40
Crystal size (mm)	0.55 × 0.1 × 0.05

Data collection	
Diffractometer	Bruker *SMART* 6000
Absorption correction	Multi-scan (*SADABS*; Bruker, 2003 ▶)
*T*_min_, *T*_max_	0.695, 0.887
No. of measured, independent and observed [*I* > 2σ(*I*)] reflections	13302, 2723, 2053
*R*_int_	0.093
(sin θ/λ)_max_ (Å^−1^)	0.614

Refinement	
*R*[*F* ^2^ > 2σ(*F* ^2^)], *wR*(*F* ^2^), *S*	0.064, 0.171, 1.06
No. of reflections	2723
No. of parameters	212
H-atom treatment	H-atom parameters constrained
Δρ_max_, Δρ_min_ (e Å^−3^)	0.49, −0.52

Computer programs: *SMART* and *SAINT* (Bruker, 2003 ▶), *SHELXS97* and *SHELXL97* (Sheldrick, 2008 ▶) and *OLEX2* (Dolomanov *et al.*, 2009 ▶).

## supplementary crystallographic information

### Crystal data

**Table:**

C_17_H_15_ClN_2_O_2_	*Z* = 2
*M**_r_* = 314.76	*F*(000) = 328
Triclinic, *P*1	*D*_x_ = 1.439 Mg m^−^^3^
*a* = 5.6966 (6) Å	Melting point: 467(1) K
*b* = 10.6852 (13) Å	Cu *K*α radiation, λ = 1.54178 Å
*c* = 12.7750 (17) Å	µ = 2.40 mm^−^^1^
α = 101.573 (7)°	*T* = 100 K
β = 100.620 (7)°	Plate, colourless
γ = 101.311 (6)°	0.55 × 0.1 × 0.05 mm
*V* = 726.47 (15) Å^3^	

### Data collection

**Table:**

Bruker SMART 6000 diffractometer	2053 reflections with *I* > 2σ(*I*)
Radiation source: fine-focus sealed tube	*R*_int_ = 0.093
Graphite monochromator	θ_max_ = 71.2°, θ_min_ = 3.6°
Absorption correction: multi-scan (*SADABS*; Bruker, 2003)	*h* = −6→6
*T*_min_ = 0.695, *T*_max_ = 0.887	*k* = −13→13
13302 measured reflections	*l* = −13→14
2723 independent reflections	

### Refinement

**Table:**

Refinement on *F*^2^	Primary atom site location: structure-invariant direct methods
Least-squares matrix: full	Secondary atom site location: difference Fourier map
*R*[*F*^2^ > 2σ(*F*^2^)] = 0.064	Hydrogen site location: inferred from neighbouring sites
*wR*(*F*^2^) = 0.171	H-atom parameters constrained
*S* = 1.06	*w* = 1/[σ^2^(*F*_o_^2^) + (0.0732*P*)^2^ + 0.2182*P*] where *P* = (*F*_o_^2^ + 2*F*_c_^2^)/3
2723 reflections	(Δ/σ)_max_ < 0.001
212 parameters	Δρ_max_ = 0.49 e Å^−^^3^
0 restraints	Δρ_min_ = −0.52 e Å^−^^3^

### Special details

**Table:**

Geometry. All e.s.d.'s (except the e.s.d. in the dihedral angle between two l.s. planes) are estimated using the full covariance matrix. The cell e.s.d.'s are taken into account individually in the estimation of e.s.d.'s in distances, angles and torsion angles; correlations between e.s.d.'s in cell parameters are only used when they are defined by crystal symmetry. An approximate (isotropic) treatment of cell e.s.d.'s is used for estimating e.s.d.'s involving l.s. planes.
Refinement. Refinement of *F*^2^ against ALL reflections. The weighted *R*-factor *wR* and goodness of fit *S* are based on *F*^2^, conventional *R*-factors *R* are based on *F*, with *F* set to zero for negative *F*^2^. The threshold expression of *F*^2^ > σ(*F*^2^) is used only for calculating *R*-factors(gt) *etc*. and is not relevant to the choice of reflections for refinement. *R*-factors based on *F*^2^ are statistically about twice as large as those based on *F*, and *R*- factors based on ALL data will be even larger.

### Fractional atomic coordinates and isotropic or equivalent isotropic displacement parameters (Å^2^)

**Table:**

	*x*	*y*	*z*	*U*_iso_*/*U*_eq_	
C1	0.3159 (5)	0.3078 (3)	0.0427 (3)	0.0335 (7)	
C2	0.3044 (5)	0.3400 (3)	0.1510 (3)	0.0321 (7)	
H2	0.161 (7)	0.3085 (15)	0.1716 (10)	0.039*	
C3	0.5098 (5)	0.4202 (3)	0.2295 (3)	0.0307 (6)	
H3	0.5032 (6)	0.4434 (11)	0.305 (3)	0.037*	
C4	0.7274 (5)	0.4666 (3)	0.1972 (3)	0.0300 (6)	
C5	0.7338 (6)	0.4289 (3)	0.0867 (3)	0.0334 (7)	
H5	0.868 (7)	0.4554 (14)	0.0670 (11)	0.040*	
C6	0.5296 (6)	0.3501 (3)	0.0079 (3)	0.0335 (7)	
H6	0.5348 (6)	0.3261 (12)	−0.066 (3)	0.040*	
C7	0.9528 (5)	0.5525 (3)	0.2711 (3)	0.0318 (7)	
H7	1.080 (6)	0.5715 (10)	0.2403 (15)	0.038*	
C8	0.9363 (6)	0.6628 (3)	0.5421 (3)	0.0331 (7)	
C9	1.2007 (6)	0.7358 (3)	0.5523 (3)	0.0348 (7)	
H9A	1.2191 (8)	0.826 (3)	0.5724 (7)	0.042*	
H9B	1.309 (3)	0.7121 (7)	0.6044 (16)	0.042*	
C10	1.2471 (5)	0.6936 (3)	0.4386 (3)	0.0328 (7)	
H10	1.362 (5)	0.644 (2)	0.4414 (3)	0.039*	
C11	1.3200 (5)	0.8005 (3)	0.3817 (3)	0.0310 (7)	
C12	1.2013 (5)	0.9032 (3)	0.3819 (3)	0.0343 (7)	
H12	1.076 (6)	0.9091 (4)	0.4234 (18)	0.041*	
C13	1.2591 (6)	0.9962 (3)	0.3244 (3)	0.0349 (7)	
H13	1.178 (4)	1.065 (3)	0.3263 (3)	0.042*	
C14	1.4372 (5)	0.9886 (3)	0.2629 (3)	0.0324 (7)	
C15	1.5645 (6)	0.8904 (3)	0.2644 (3)	0.0332 (7)	
H15	1.688 (6)	0.8869 (3)	0.2269 (17)	0.040*	
C16	1.5032 (5)	0.7974 (3)	0.3235 (3)	0.0323 (7)	
H16	1.590 (4)	0.729 (3)	0.3241 (3)	0.039*	
C17	1.6705 (7)	1.0860 (4)	0.1506 (3)	0.0438 (8)	
H17A	1.641 (3)	0.998 (3)	0.095 (2)	0.066*	
H17B	1.833 (4)	1.103 (3)	0.2060 (15)	0.066*	
H17C	1.677 (4)	1.159 (3)	0.110 (2)	0.066*	
Cl1	0.05505 (14)	0.21060 (9)	−0.05673 (7)	0.0434 (3)	
N1	0.9930 (4)	0.6049 (2)	0.3753 (2)	0.0287 (6)	
N2	0.8252 (5)	0.5891 (3)	0.4379 (2)	0.0306 (6)	
O1	0.8327 (4)	0.6705 (3)	0.6184 (2)	0.0432 (6)	
O2	1.4761 (4)	1.0831 (2)	0.2062 (2)	0.0382 (5)	

### Atomic displacement parameters (Å^2^)

**Table:**

	*U*^11^	*U*^22^	*U*^33^	*U*^12^	*U*^13^	*U*^23^
C1	0.0211 (14)	0.0340 (16)	0.0422 (19)	0.0060 (12)	0.0036 (12)	0.0064 (13)
C2	0.0210 (14)	0.0334 (15)	0.0425 (19)	0.0047 (11)	0.0088 (12)	0.0111 (13)
C3	0.0239 (14)	0.0326 (15)	0.0347 (18)	0.0048 (12)	0.0077 (12)	0.0077 (12)
C4	0.0240 (14)	0.0277 (14)	0.0389 (18)	0.0045 (11)	0.0094 (12)	0.0095 (12)
C5	0.0215 (14)	0.0340 (16)	0.0456 (19)	0.0043 (12)	0.0111 (12)	0.0113 (13)
C6	0.0286 (15)	0.0367 (16)	0.0357 (18)	0.0082 (12)	0.0098 (12)	0.0077 (13)
C7	0.0199 (14)	0.0322 (15)	0.0438 (19)	0.0040 (11)	0.0115 (12)	0.0091 (13)
C8	0.0293 (16)	0.0328 (15)	0.0389 (18)	0.0057 (12)	0.0096 (13)	0.0128 (12)
C9	0.0259 (15)	0.0343 (16)	0.0419 (19)	0.0024 (12)	0.0065 (12)	0.0101 (13)
C10	0.0185 (14)	0.0316 (15)	0.0463 (19)	0.0029 (12)	0.0056 (12)	0.0100 (13)
C11	0.0181 (13)	0.0316 (15)	0.0383 (17)	0.0019 (11)	0.0028 (11)	0.0049 (12)
C12	0.0200 (14)	0.0408 (17)	0.0414 (19)	0.0050 (12)	0.0092 (12)	0.0089 (13)
C13	0.0239 (15)	0.0328 (16)	0.047 (2)	0.0074 (12)	0.0056 (13)	0.0089 (13)
C14	0.0226 (14)	0.0308 (15)	0.0401 (18)	0.0003 (11)	0.0039 (12)	0.0094 (12)
C15	0.0219 (14)	0.0335 (16)	0.0424 (18)	0.0025 (12)	0.0086 (12)	0.0088 (13)
C16	0.0166 (13)	0.0340 (16)	0.0443 (19)	0.0042 (11)	0.0058 (12)	0.0083 (13)
C17	0.0370 (18)	0.048 (2)	0.050 (2)	0.0084 (15)	0.0136 (15)	0.0193 (16)
Cl1	0.0231 (4)	0.0522 (5)	0.0450 (5)	0.0033 (3)	0.0050 (3)	−0.0024 (3)
N1	0.0187 (12)	0.0307 (13)	0.0374 (15)	0.0043 (9)	0.0089 (10)	0.0094 (10)
N2	0.0230 (12)	0.0348 (13)	0.0355 (15)	0.0049 (10)	0.0108 (10)	0.0103 (10)
O1	0.0362 (13)	0.0502 (14)	0.0420 (14)	0.0023 (10)	0.0156 (10)	0.0105 (10)
O2	0.0303 (11)	0.0371 (12)	0.0484 (14)	0.0055 (9)	0.0088 (9)	0.0161 (10)

### Geometric parameters (Å, º)

**Table:**

C1—C2	1.374 (5)	C10—H10	0.9240
C1—C6	1.395 (4)	C10—C11	1.506 (4)
C1—Cl1	1.752 (3)	C10—N1	1.539 (4)
C2—H2	0.9276	C11—C12	1.398 (5)
C2—C3	1.392 (4)	C11—C16	1.389 (4)
C3—H3	0.9509	C12—H12	0.9660
C3—C4	1.408 (4)	C12—C13	1.375 (5)
C4—C5	1.398 (5)	C13—H13	0.9441
C4—C7	1.458 (4)	C13—C14	1.398 (5)
C5—H5	0.8642	C14—C15	1.388 (5)
C5—C6	1.385 (5)	C14—O2	1.363 (4)
C6—H6	0.9337	C15—H15	0.9229
C7—H7	0.8959	C15—C16	1.394 (5)
C7—N1	1.296 (4)	C16—H16	0.9566
C8—C9	1.523 (4)	C17—H17A	1.0168
C8—N2	1.366 (4)	C17—H17B	1.0168
C8—O1	1.227 (4)	C17—H17C	1.0168
C9—H9A	0.9313	C17—O2	1.420 (4)
C9—H9B	0.9313	N1—N2	1.362 (3)
C9—C10	1.519 (5)		
			
C2—C1—C6	122.3 (3)	C11—C10—H10	109.6
C2—C1—Cl1	119.6 (2)	C11—C10—N1	109.6 (3)
C6—C1—Cl1	118.0 (3)	N1—C10—H10	109.6
C1—C2—H2	120.2	C12—C11—C10	121.6 (3)
C1—C2—C3	119.6 (3)	C16—C11—C10	120.7 (3)
C3—C2—H2	120.2	C16—C11—C12	117.7 (3)
C2—C3—H3	120.2	C11—C12—H12	119.4
C2—C3—C4	119.7 (3)	C13—C12—C11	121.2 (3)
C4—C3—H3	120.2	C13—C12—H12	119.4
C3—C4—C7	124.9 (3)	C12—C13—H13	119.9
C5—C4—C3	119.0 (3)	C12—C13—C14	120.3 (3)
C5—C4—C7	116.1 (3)	C14—C13—H13	119.9
C4—C5—H5	119.2	C15—C14—C13	119.7 (3)
C6—C5—C4	121.7 (3)	O2—C14—C13	115.8 (3)
C6—C5—H5	119.2	O2—C14—C15	124.5 (3)
C1—C6—H6	121.1	C14—C15—H15	120.5
C5—C6—C1	117.7 (3)	C14—C15—C16	119.0 (3)
C5—C6—H6	121.1	C16—C15—H15	120.5
C4—C7—H7	115.7	C11—C16—C15	122.0 (3)
N1—C7—C4	128.5 (3)	C11—C16—H16	119.0
N1—C7—H7	115.7	C15—C16—H16	119.0
N2—C8—C9	112.6 (3)	H17A—C17—H17B	109.5
O1—C8—C9	123.9 (3)	H17A—C17—H17C	109.5
O1—C8—N2	123.5 (3)	H17B—C17—H17C	109.5
C8—C9—H9A	110.8	O2—C17—H17A	109.5
C8—C9—H9B	110.8	O2—C17—H17B	109.5
H9A—C9—H9B	108.9	O2—C17—H17C	109.5
C10—C9—C8	104.7 (3)	C7—N1—C10	120.2 (2)
C10—C9—H9A	110.8	C7—N1—N2	125.3 (3)
C10—C9—H9B	110.8	N2—N1—C10	114.5 (2)
C9—C10—H10	109.6	N1—N2—C8	107.3 (2)
C9—C10—N1	100.9 (2)	C14—O2—C17	117.6 (3)
C11—C10—C9	117.1 (3)		
			
C1—C2—C3—C4	−0.4 (5)	C10—C11—C16—C15	176.0 (3)
C2—C1—C6—C5	−1.0 (5)	C10—N1—N2—C8	1.7 (3)
C2—C3—C4—C5	−1.3 (5)	C11—C10—N1—C7	53.0 (4)
C2—C3—C4—C7	179.0 (3)	C11—C10—N1—N2	−127.0 (3)
C3—C4—C5—C6	1.8 (5)	C11—C12—C13—C14	0.8 (5)
C3—C4—C7—N1	−2.9 (5)	C12—C11—C16—C15	−1.8 (5)
C4—C5—C6—C1	−0.7 (5)	C12—C13—C14—C15	−3.1 (5)
C4—C7—N1—C10	180.0 (3)	C12—C13—C14—O2	178.2 (3)
C4—C7—N1—N2	−0.1 (5)	C13—C14—C15—C16	3.0 (5)
C5—C4—C7—N1	177.4 (3)	C13—C14—O2—C17	174.5 (3)
C6—C1—C2—C3	1.6 (5)	C14—C15—C16—C11	−0.5 (5)
C7—C4—C5—C6	−178.4 (3)	C15—C14—O2—C17	−4.1 (5)
C7—N1—N2—C8	−178.3 (3)	C16—C11—C12—C13	1.7 (5)
C8—C9—C10—C11	121.5 (3)	Cl1—C1—C2—C3	−178.3 (2)
C8—C9—C10—N1	2.6 (3)	Cl1—C1—C6—C5	178.8 (2)
C9—C8—N2—N1	0.2 (3)	N1—C10—C11—C12	70.4 (4)
C9—C10—C11—C12	−43.7 (4)	N1—C10—C11—C16	−107.3 (3)
C9—C10—C11—C16	138.5 (3)	N2—C8—C9—C10	−2.0 (4)
C9—C10—N1—C7	177.1 (3)	O1—C8—C9—C10	178.9 (3)
C9—C10—N1—N2	−2.8 (3)	O1—C8—N2—N1	179.4 (3)
C10—C11—C12—C13	−176.1 (3)	O2—C14—C15—C16	−178.4 (3)

### Hydrogen-bond geometry (Å, º)

Cg is the centroid of the C1–C6 ring.

**Table:**

*D*—H···*A*	*D*—H	H···*A*	*D*···*A*	*D*—H···*A*
C3—H3···N2	0.96 (4)	2.31 (3)	2.934 (4)	122 (1)
C3—H3···O1^i^	0.96 (4)	2.52 (2)	3.152 (4)	124 (1)
C17—H17*C*···*Cg*^ii^	1.02 (3)	2.73 (3)	3.551 (4)	138 (2)

Symmetry codes: (i) −*x*+1, −*y*+1, −*z*+1; (ii) *x*+1, *y*+1, *z*.
